# The Dynamics of Germinal Centre Selection as Measured by Graph-Theoretical Analysis of Mutational Lineage Trees

**DOI:** 10.1080/10446670310001593541

**Published:** 2002-12

**Authors:** Deborah K. Dunn-Walters, Alex Belelovsky, Hanna Edelman, Monica Banerjee, Ramit Mehr

**Affiliations:** 1 Departments of Immunobiology and Histopathology GKT Medical School London UK; 2 Department of Mathematics and Computer Sciences Bar-Ilan University Ramat-Gan 52900 Israel; 3 Faculty of Life Sciences Bar-Ilan University Ramat-Gan 52900 Israel

## Abstract

We have developed a rigorous graph-theoretical algorithm for quantifying the shape properties of mutational lineage trees. We show that information about the dynamics of hypermutation and antigen-driven clonal selection during the humoral immune response is contained in the shape of mutational lineage trees deduced from the responding clones. Age and tissue related differences in the selection process can be studied using this method. Thus, tree shape analysis can be used as a means of elucidating humoral immune response dynamics in various situations.


*Developmental Immunology*, December 2002 Vol. 9 (4), pp. 233–243

Taylor & Francis healthsciences

### The Dynamics of Germinal Centre Selection as Measured by Graph-Theoretical Analysis of Mutational Lineage Trees*

DEBORAH K. DUNN-WALTERS[a,†], ALEX BELELOVSKY[b], HANNA EDELMAN[c], MONICA BANERJEE[a] and RAMIT MEHR[c]

[a]*Departments of Immunobiology and Histopathology, GKT Medical School, London, UK;* [b]*Department of Mathematics and Computer Sciences, Bar-Ilan University, Ramat-Gan 52900, Israel;* [c]*Faculty of Life Sciences, Bar-Ilan University, Ramat-Gan 52900, Israel*

We have developed a rigorous graph-theoretical algorithm for quantifying the shape properties of mutational lineage trees. We show that information about the dynamics of hypermutation and antigen-driven clonal selection during the humoral immune response is contained in the shape of mutational lineage trees deduced from the responding clones. Age and tissue related differences in the selection process can be studied using this method. Thus, tree shape analysis can be used as a means of elucidating humoral immune response dynamics in various situations.

*Keywords*: B lymphocytes; Somatic hypermutation; Germinal centre; Antibodies

### INTRODUCTION

Memory B lymphocyte generation involves affinity maturation of the cells' antigen receptors, based on somatic hypermutation of receptor genes and antigen-driven selection of the resulting mutants (Kelsoe, 1996; Wabl *et al.*, 1999; Neuberger *et al.*, 2000; Diaz and Casali, 2002). Hypermutation of immunoglobulin variable region genes is several orders of magnitude faster than normal somatic mutation, and there is evidence that hypermutation is generated by a different mechanism than that of normal somatic mutation (Winter and Gearhart, 1998; Neuberger *et al.*, 1998; Cowell and Kepler, 2000). The exact mechanism of somatic hypermutation is yet unknown, although it has been shown to depend on transcription, activation-induced cytidine deaminase (AID) and DNA mismatch repair mechanisms. It is thought that the mechanism is related to that of class switch recombination (Honjo, 2002). Many questions are still open, such as how somatic hypermutation is triggered and regulated; whether immune complexes play a role (Song *et al.*, 1998; 1999) and how the processes of hypermutation and selection interact to shape the memory B cell repertoire.

Theoretical approaches utilized so far in the study of affinity maturation include the analysis of the frequencies of specific types of mutations (Dunn-Walters *et al.*, 1998; Dorner *et al.*, 1998; Spencer *et al.*, 1999; Oprea and Kepler, 1999; Kim *et al.*, 1999; Foster *et al.*, 1999; Monson *et al.*, 2000; Michael *et al.*, 2002), and mathematical models exploring the dynamical interactions between somatic hypermutation and clonal selection (Sulzer *et al.*, 1993; Kepler and Perelson, 1993; Oprea and Perelson, 1997; Shlomchik *et al.*, 1998; Shannon and Mehr, 1999; Kesmir and de Boer, 1999). In this study we present a new approach—the analysis of the shapes of mutational lineage trees.

The generation of "lineage trees" or "dendrograms" to visualize the lineage relationships of B cell mutants in the GCs has been used in the past to confirm the role of the GC as the location of somatic hypermutation (Kocks and Rajewsky, 1988; Manser, 1989; Jacob *et al.*, 1991), to identify lineage relationships between cells from independent GCs (Vora *et al.*, 1999) or different tissues (Dunn-Walters *et al.*, 1997a,b) and from additional processes of diversification such as gene conversion in the rabbit (Seghal *et al.*, 1998; Schiaffella *et al.*, 1999; Seghal *et al.*, 2000; Seghal *et al.*, 2002). The experimentally generated lineage trees reflect multiple rounds of mutations for each germline V gene that participated in the primary response. We believe that much information about the dynamics of antigen-driven clonal selection during the immune response is contained in the shape of lineage trees deduced from the final responding clones (Shannon and Mehr, 1999). For example, trees generated from clones during the peak of the primary response are much more "bushy" (Jacob and Kelsoe, 1992), but trees become less "bushy" as the response progresses (Jacob *et al.*, 1993). The "pruned" shape of these trees has been referred to as evidence for the destructive character of somatic hypermutation. Other examples of lineage trees

*Presented at the Proceedings of the 4th Germinal Center Conference, June 2002, Groningen, The Netherlands.

†Corresponding author. Tel.: +44-20-7955-4467. Fax: +44-20-7955-4961. E-mail: deborah.dunn-walters@kcl.ac.uk

ISSN 1044-6672 print © 2002 Taylor & Francis Ltd
DOI: 10.1080/10446670310001593541

drawn to illustrate various aspects of the germinal centre reaction, or differences in this reaction under varying circumstances, abound in the literature. So far, however, lineage tree classification has been based only on a qualitative, intuitive assessment of the most obvious shape characteristics. Hence we set out to explore whether the information embedded in the mathematical shape characteristics of lineage trees can in any way be quantifiable, and whether it can be shown to correlate with the dynamics of the underlying immune response.

The objective of the present study was to develop a rigorous computer-aided algorithm for extracting the information contained in lineage trees, using the tools of mathematical graph theory. The algorithm we developed is composed of a module that characterizes trees according to their various graph-theoretical measures, and another module for finding correlations between these measures and the dynamical parameters of the GC response that generated the trees. Note that, for the purpose of our analysis, we are not interested in the properties of the individual cells or clones represented by the lineage tree, but rather in the overall characteristics of the lineage tree as a graphical entity. We demonstrate in the following that the information extracted using our algorithm is indeed valuable in revealing the dynamics of hypermutation and antigen-driven selection in germinal centres.

### RESULTS

### Tree Similarity and Size Scaling

Measurement of published lineage trees reveals several interesting details about our method, even though published data are too scarce for statistical analyses (only 1–2 trees are usually published as an illustration). First, when two trees develop from the same germline gene under similar conditions (two different GCs in the same response (Jacob *et al.*, 1991), the trees are indeed similar in all aspects measured (Fig. 1a,b). While the profiles of the two trees are similar, it is obvious that most properties vary with tree size, e.g. tree II is slightly larger in most measured properties than tree I. In order to properly compare trees, we must distinguish between two types of tree properties: those that are independent of tree size and those that correlate with tree size. Examples of size-independent properties are: root degree, maximum or average outgoing degree and maximum or average distance between a leaf to the nearest split node. Examples of size-dependent properties are: the number of internal or pass-through nodes, the maximum or average path length (from root to leaf), trunk length, etc. We set out to examine whether scaling these properties by tree size gives a better measure of tree similarity or difference.

As previously mentioned, there are two different measures of tree size that could be used to scale the size-dependent tree properties. The total number of nodes seems to be the most natural measure. The number of internal nodes, or the number of pass-through nodes (which are a subset of all internal nodes), correlates well with the total number of nodes. Scaling by the number of leaves is more problematic, as it is highly sensitive to the sampling process, that is, to the number of cells from any given clone that were found in the experiment. It is also highly sensitive to the particular germline gene involved, as different germline genes differ in their potential for improvement by mutation (Shannon and Mehr, 1999). Additionally, as the response progresses, the number of nodes per leaf may grow, as the tree gets longer by the addition of mutations, and more "pruned" through the action of selection (see next section). Hence scaling should be done with care. However, when two trees are generated from the same germline gene in the same response, as in the case shown above, we find that they are very similar in all their scaled size-dependent properties, whether scaling is done by total number of nodes or by number of leaves (Fig. 1c,d). Similar results were obtained for the two trees published in (Jacob and Kelsoe, 1992).

### Trees Grow and are "Pruned" as the Response Progresses

When trees are taken from a response to the same antigen, but in different times during the response (Jacob *et al.*, 1993), the trees seem to gradually change towards a longer, more pruned, shape. There is a consistent change in several tree parameters (Fig. 2). For example, the number of leaves not only does not increase, but actually decreases with time. This is probably due to the effect of selection that "prunes" branches corresponding to useless or lower-affinity mutants. Two measures of tree "bushiness", which is expected to decrease with time as a result of selection, also decrease- the maximum and average (excluding pass-through nodes) outgoing degree of a node.

On the other hand, trees from the primary response are very similar in structure to those from the secondary response, at least in the one published example we analysed (Vora *et al.*, 1999). Our measurements show that the two trees are similar in every aspect (Fig. 3).

### Our Analysis can Distinguish between Trees from Different Sources

Trees from GCs from spleen and Peyer's patches of young and old human patients (Banerjee *et al.*, 2002) were analysed by our algorithm. Data clearly shows that the trees from the spleen show signs of having been subject to stronger selective forces. Both the maximum and average outgoing degree were smaller in the spleen than in the Peyer's patches, indicating a more bushy, and less selected, response in the Peyer's patch. Similarly, both the maximum and average distance from last split node to leaf were increased in the spleen compared to the Peyer's patches (Fig. 4). This algorithm also showed some age-related differences in selection that concurred

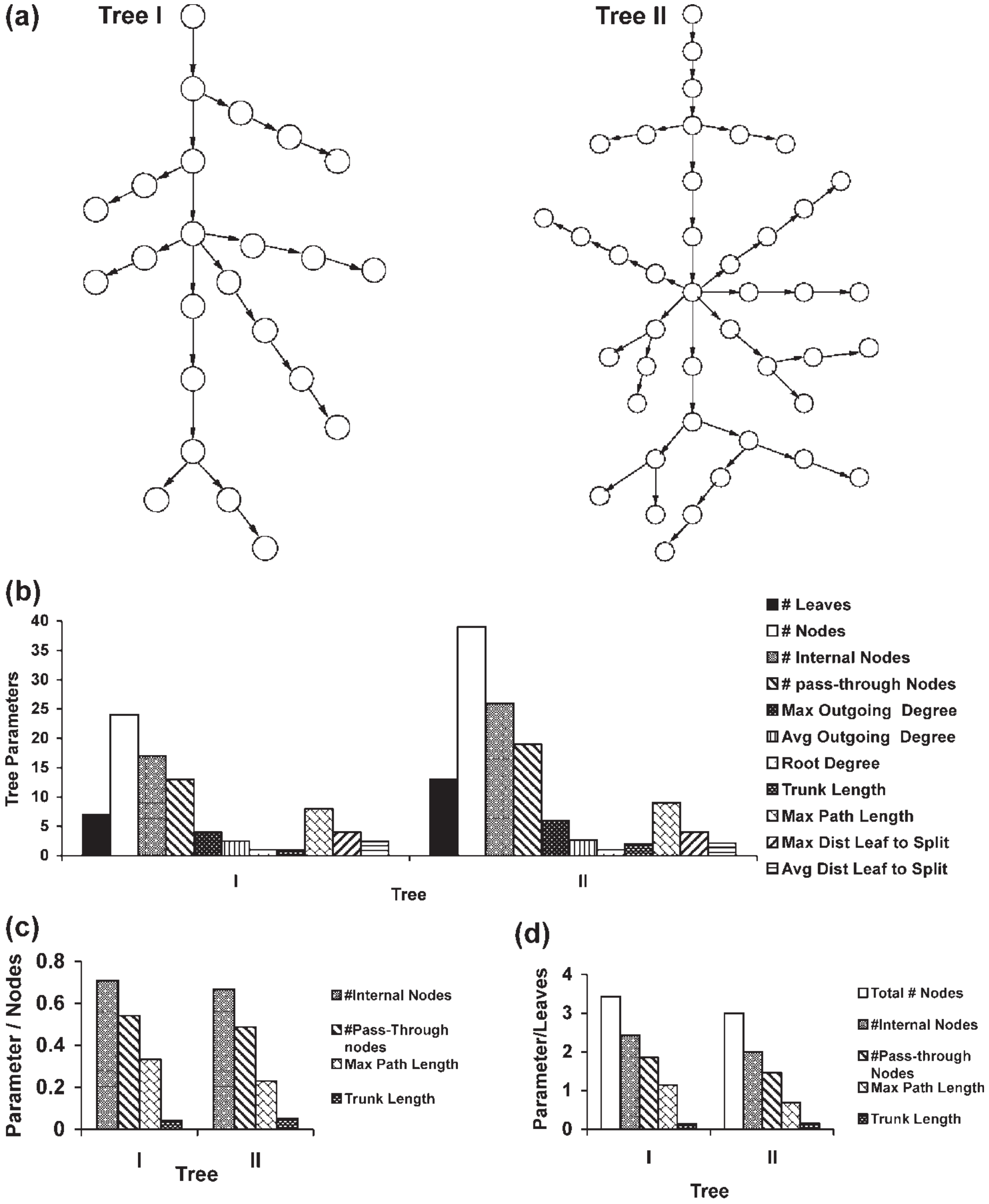

FIGURE 1 Similarity of two trees obtained from two different GCs in the same response. (a) Trees from (Jacob *et al.*, 1991), drawn such that each mutation is shown as a separate node. (b) A comparison of the "profiles" (selected graphical properties) of the trees. The trees are very similar in all their scaled size-dependent properties, whether scaling is done by total number of nodes (c) or by number of leaves (d).

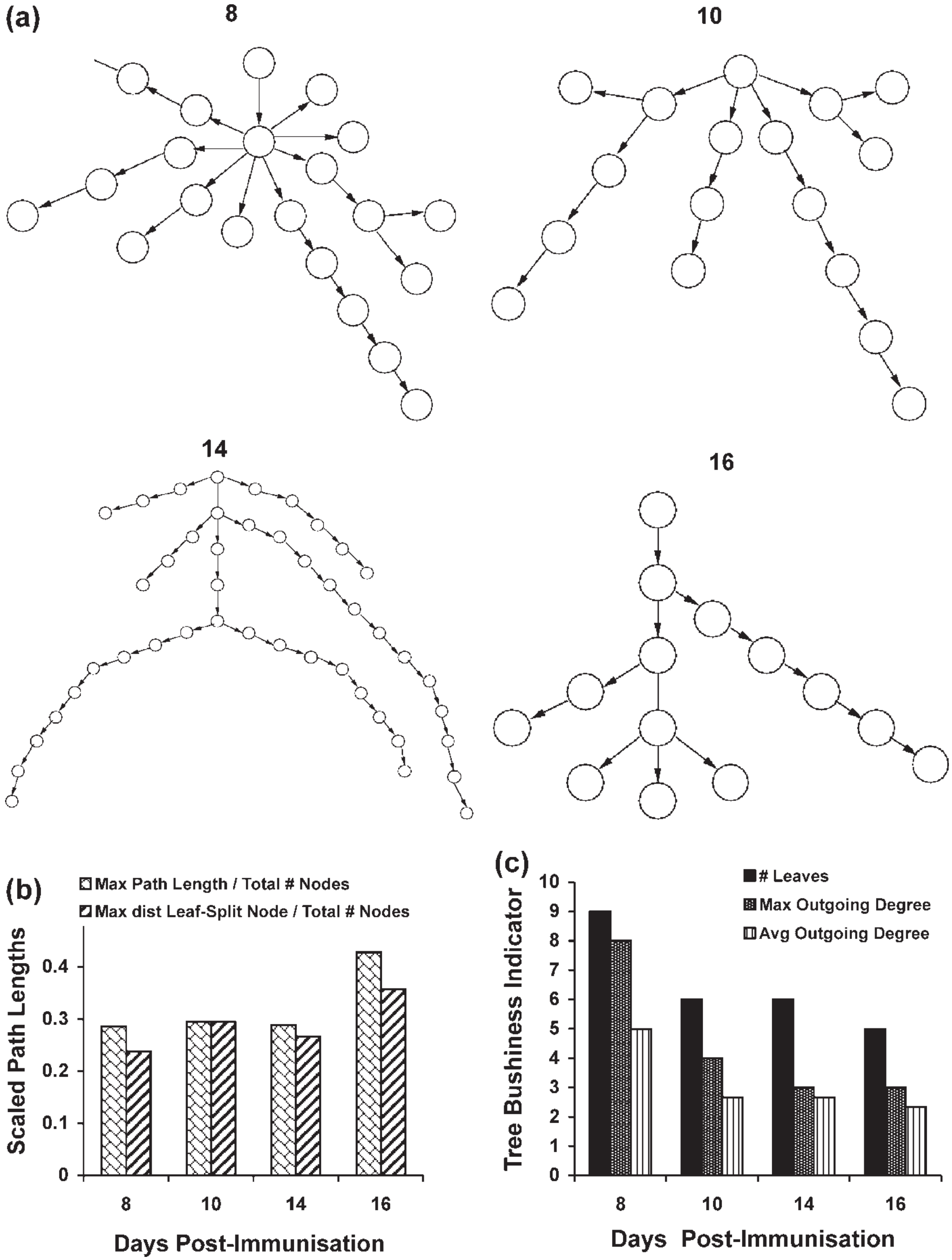

FIGURE 2 Changes in tree shape in the course of the immune response (Jacob *et al.*, 1993). (a) The trees. (b) Scaled path lengths as function of time in the response. (c) Three measures of tree "bushiness" as function of time in the response.

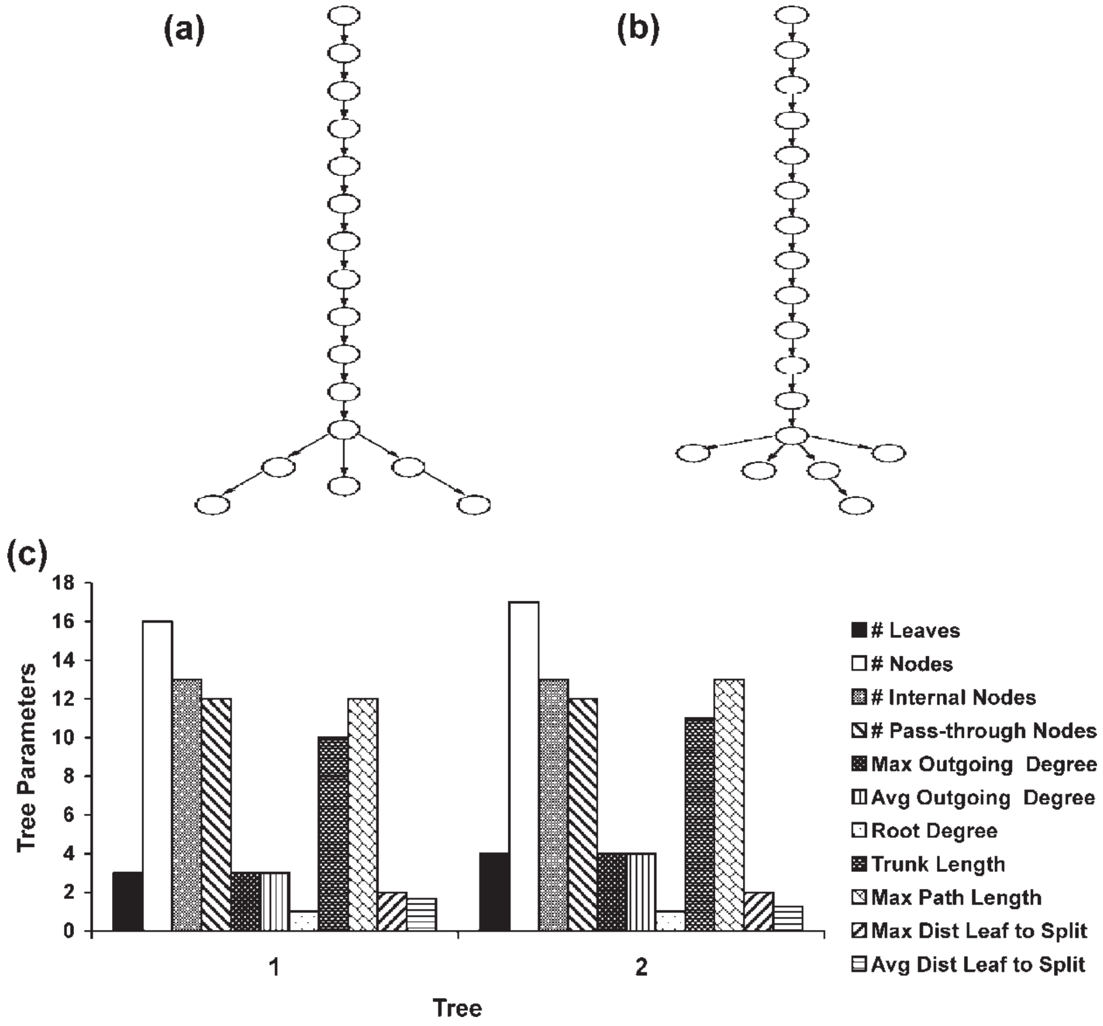

FIGURE 3 Comparison (c) between trees from a primary (a) and secondary (b) response (Vora *et al.*, 1999), showing their similarity.

with other measures used to analyse the data (Banerjee *et al.*, 2002).

### Analysis of Simulated Trees—"Tree Generator"

The analysis of published trees indicated that graphical tree parameters may indeed correlate with the biological parameters of the germinal centre response. However, there is not enough published data for conclusive analysis. In order to extract possible correlations between the graphical parameters describing a tree and the biological parameters describing the corresponding affinity maturation process, we would have to statistically analyse a significant number of trees with *a priori* known biological parameters. There are only a few tens of trees obtained experimentally and published, which are not sufficient for this purpose, and even if their numbers were sufficient, not all the biological parameters are known for experimental trees. Hence we decided to define and implement a simple simulation of the humoral immune response, which will allow us to control mutation and selection parameters, and produce lineage trees.

### Simulation Parameters and their Effect on Tree Shape

This section summarizes the "biological" parameters controlling tree generation by our simulation. Varying these parameters enables us to produce trees corresponding to different values and then analyse the subsequent change in tree shape. We chose parameter values which model the affinity maturation process in the most realistic way, based on experimental data, while keeping the model as simple as possible. Simulation parameters are given in Table I.

The *mutation mode* parameter denotes how the simulation interprets the mutation rate parameter. Its values are Bit (the default) and Div. In Bit mode, the mutation rate is the probability of a single bit in the receptor string to mutate. In Div mode, the mutation rate is the fixed percentage of bits that mutate per division.

The *selection mode* parameter defines the method of selection, and how the simulation uses the *selection threshold* parameter, which denotes the minimum affinity required for a cell to survive selection. In Abs (absolute) selection mode, the selection threshold remains constant. In Rel (relative) selection mode, the selection threshold

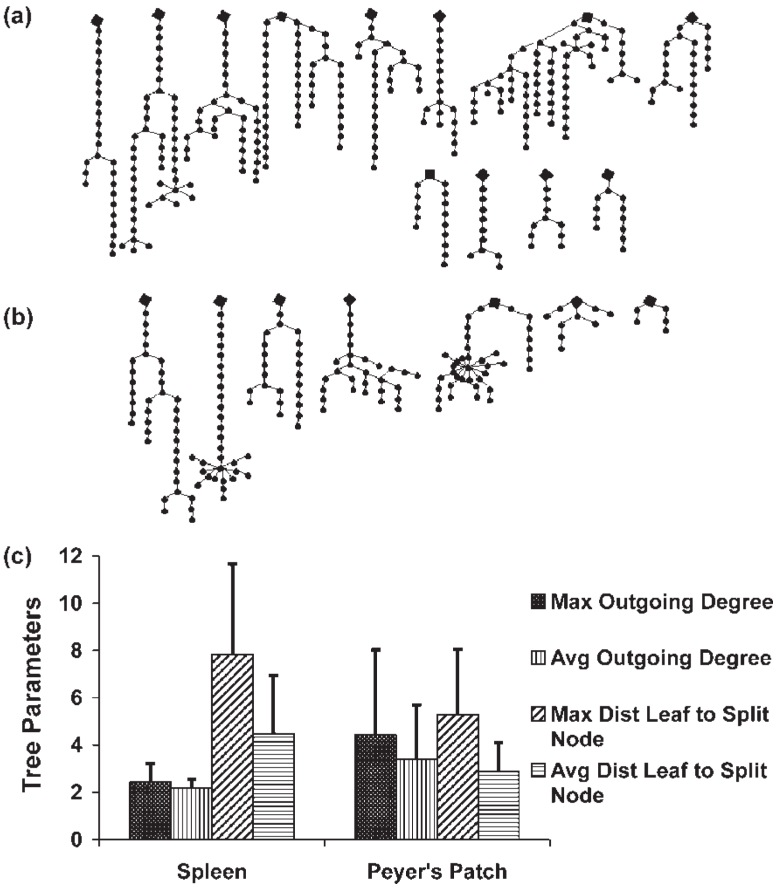

FIGURE 4 Comparison (c) between trees isolated from the germinal centres of human spleen (a) and Peyer's patches (b) (Banerjee *et al.*, 2002). The *p* values for the differences between the two tissues in maximum and average outgoing degree and maximum and average distance from leaf to split node are 0.04, 0.04, 0.07 and 0.07, respectively.

corresponds to the average affinity in the population; however, in the first generation the pre-defined selection threshold is used.

The *selection rate* parameter defines the probability for a cell to undergo selection in each generation. That is, $(selection\ rate)^{-1}$ denotes the maximal number of mutations between two consequent selection events.

The *selection start* parameter defines the time selection begins to operate (in number of generations since the simulation started).

The values chosen for each of the above parameters (Table I) yielded 1920 different parameter sets. Each different parameter set was used in five different simulations, with five different random number generator seeds. Some simulations yielded more than one tree and in some cases no trees were generated (when all cells died before the end of the simulation). The total number of trees generated was 8300. Several features, which indicated that tree shapes indeed reflect the dynamics of the response, were observed during simulation development and tree generation, as follows.

1. As expected, the relative selection mode is more effective than absolute selection—in the case of relative selection, the population contains fewer cells and their average affinity is higher than in the case of absolute selection, in which there is no way to develop nor to kill the cells with relatively low affinity obtained at the first steps of the simulation. The interesting point is that this is reflected in tree shapes. Trees obtained in relative selection mode have fewer branches and their average length is higher than trees obtained in absolute selection mode. For example, the average number of leaves for all 5175 trees obtained in the absolute selection mode is $4648 \pm 4701$, and the average distance from a leaf back to the last split is only

TABLE I Parameter values in the tree generator simulations

| Parameter | Values |
|---|---|
| Number of clones | 1 or 5 |
| Initial number of cells | 10 |
| Is the population limited? | Yes or No |
| Maximal population size | 10,000 |
| Initial amount of antigen | $10^5$ units |
| Mutation mode | Bitwise or Div. |
| Mutation rate | 0.002, 0.004, 0.006, 0.008 |
| Selection Mode | Abs, Rel |
| Selection threshold | 0.5, 0.6, 0.7, 0.8, 0.9 |
| Selection rate | 0.25, 0.3, 0.5, 1.0 |
| Selection start | 1, 10 |

All rates are per a simulation step representing 6 h.

$1.85 \pm 0.55$ nodes, while for the 3125 trees obtained in the relative selection mode, the average number of leaves is $584 \pm 911$ and the average distance from a leaf back to the last split is $2.25 \pm 4.50$. The trees are larger than published experimental trees because (i) here we have the full tree and not just a sample of it in each case and (ii) not all parameter sets reflect biologically relevant parameter regimes. However, the fact that the standard deviations in the number of nodes (and the leaf to last split distance in the relative selection mode) are much larger than the means shows that the means were at the lower end of the range, that is, there were many more smaller trees than larger trees. The majority of trees having non-zero and long trunks were obtained in relative selection mode as well: there were 3787 trees with no trunk among the trees obtained in the absolute selection mode, and none among the trees obtained in the relative selection mode; there were only 1201 out of 5175 trees with trunk length $\geq 3$ in the absolute selection mode, compared with 2056 out of the 3125 trees obtained in the relative selection mode.

2. In the case of "effective" (relative mode) selection, population size is relatively small throughout the simulation, and therefore antigen consumption is lower. Thus, in most cases of absolute selection, the antigen was totally exhausted before the simulation has reached 90 time steps, while in most cases of relative selection, the simulation stopped on the 90th step with a certain amount of remaining antigen.
3. The probability of getting no tree (all cells dying by the end of the simulation) grows with mutation rate. For a mutation rate of 0.008 (4 bits flipped per generation), all cells died regardless of the values of all other parameters. For a mutation rate of 0.002 (1 bit flipped per generation), almost every simulation yielded at least one tree. Hence higher values probably reflect unrealistic mutation rates.
4. In those simulations where 5 clones were allowed to develop in parallel, at least one clone always died out. In most cases, only one or two trees (out of 5 initial clones) were generated. This is an expected result of interclonal competition.
5. In most cases, population size upon simulation completion was below 10000 cells even when an upper limit was not used (only in 0.6% of cases did the population exceed 20000 cells). In cases where the population did exceed 20000, selection was weak, and in most such cases selection mode was absolute.

### Correlations between Biological and Graphical Parameters

We proceeded to search for correlations between biological parameters and graphical ones, beginning by looking for simple (linear) one-to-one correlations between each biological parameter and each graphical one. We have found a surprisingly large number of correlations that were significant, though most of them had low correlation coefficients, most likely because of the high variability between trees in almost all parameters measured. Table II gives the one-to-one linear correlation coefficients and their $p$-values for all graphical parameters measured. It is evident that most graphical parameters correlated only with the mutation rate and with the selection threshold. The number of initial clones (1 or 5), the rate of selection (number of mutation rounds between two rounds of selection) and the time of starting the selection (in the beginning of the simulation or 10 generations later), in the ranges used in our simulations, did not correlate strongly ($|R| \leq 0.1$) with any of the graphical parameters.

Similar results were obtained with the scaled graphical parameters, whether they were scaled by the number of leaves or by the number of nodes (data not shown).

### DISCUSSION

The objective of the present study was to develop a rigorous algorithm for extracting the information contained in mutational lineage trees, which so far were only used as an illustration of the dynamics of the humoral immune response. The algorithm we developed is composed of a module that characterizes trees according to their various graph-theoretical measures, and another module for finding correlations between these measures and the dynamical parameters of the GC response which generated the trees. The measurement module alone is useful in analysis of trees from different experimental sources, in that it can show which tree properties are significantly different between trees from different experimental groups [Banerjee *et al.*, 2002]. Analysis of additional data will possibly enable us to hone this method further (see appendix).

Our statistical analysis validates our basic premise, that the shapes of lineage trees contain biological information on the dynamics of the germinal centre response that generated the trees. One may ask whether methods based on non-linear functions might have been more successful in prediction of the biological parameters from the graphical ones. We have attempted to improve our predictions by using a co-evolutionary algorithm, which allowed a population of proposed solutions (general polynomial functions of the graphical parameters) to co-evolve with a population of test cases (from the data analysed above). However, this method has not yielded better results than straightforward linear stepwise regression. We presume that the high variability of the trees in our simulated tree database—and possibly of experimentally-generated trees as well—precludes better prediction of biological parameters from the graphical ones, at least using regression methods.

Several more questions may be raised with respect to tree shape analysis. For example, there is the question of

TABLE II Linear correlations between graphical and "biological" (simulation) parameters

| Graphical parameter | Number of clones | Mutation rate | Selection threshold | Selection rate | Selection start |
|---|---|---|---|---|---|
| Number of leaves | 0.00215 | **− 0.22681** | **0.66992** | 0.00662 | − 0.0162 |
| *p* | 0.845 | **< 0.0001** | **< 0.0001** | 0.5468 | 0.1401 |
| Trunk length | − 0.04733 | **0.26044** | **− 0.51446** | − 0.01583 | 0.02102 |
| *p* | < .0001 | < *0.0001* | **< 0.0001** | 0.1495 | 0.0555 |
| Root degree | 0.00928 | **− 0.27867** | **0.78982** | 0.01528 | − 0.025 |
| *p* | 0.3979 | **< 0.0001** | **< 0.0001** | 0.1641 | 0.0228 |
| Number of internal nodes | − 0.00654 | **− 0.21114** | **0.57521** | − 0.00123 | − 0.01645 |
| *p* | 0.5516 | **< 0.0001** | **< 0.0001** | 0.9109 | 0.1341 |
| Number of pass-through nodes | − 0.00892 | **− 0.19937** | **0.53121** | − 0.00253 | − 0.01612 |
| *p* | 0.4166 | **< 0.0001** | **< 0.0001** | 0.8175 | 0.142 |
| Total number of nodes | − 0.00399 | **− 0.22163** | **0.62006** | 0.00119 | − 0.01681 |
| *p* | 0.7161 | **< 0.0001** | **< 0.0001** | 0.9135 | 0.1258 |
| Min outgoing degree | 0.00406 | 0.02308 | − 0.02837 | − 0.00367 | 0.00382 |
| *p* | 0.7116 | 0.0355 | 0.0098 | 0.7385 | 0.7277 |
| Max outgoing degree | 0.00664 | **− 0.23985** | **0.64914** | 0.01083 | − 0.02137 |
| *p* | 0.5455 | **< 0.0001** | **< 0.0001** | 0.3238 | 0.0516 |
| Avg outgoing degree | 0.03002 | **− 0.25499** | **0.56362** | 0.01949 | − 0.02061 |
| *p* | 0.0062 | **< 0.0001** | **< 0.0001** | 0.0758 | 0.0605 |
| Avg outgoing degree (no 1's) | 0.02435 | **− 0.16547** | **0.44563** | 0.01518 | − 0.01438 |
| *p* | 0.0266 | **< 0.0001** | **< 0.0001** | 0.1668 | 0.1902 |
| Min path length | − 0.0246 | **0.24389** | **− 0.57483** | − 0.01529 | 0.01353 |
| *p* | 0.025 | **< 0.0001** | **< 0.0001** | 0.1638 | 0.2178 |
| Max path length | − 0.02103 | 0.12281 | **− 0.36184** | − 0.0145 | 0.00858 |
| *p* | 0.0554 | <0001 | **< 0.0001** | 0.1867 | 0.4344 |
| Avg path length | − 0.02072 | 0.14458 | **− 0.43682** | − 0.01055 | 0.00595 |
| *p* | 0.0591 | <0.0001 | **< 0.0001** | 0.3364 | 0.5879 |
| Min dist between adjacent split nodes | 0.00198 | 0.07449 | − 0.06054 | − 0.00742 | 0.02944 |
| *p* | 0.8568 | <0.0001 | <0.0001 | 0.4991 | 0.0073 |
| Max dist between adjacent split nodes | 0.00473 | 0.05858 | − 0.19647 | − 0.0069 | 0.00239 |
| *p* | 0.6664 | <0.0001 | <0.0001 | 0.5299 | 0.8279 |
| Avg dist between adjacent split nodes | − 0.02032 | **0.19893** | **− 0.39286** | − 0.00829 | 0.02496 |
| *p* | 0.0642 | **< 0.0001** | **< 0.0001** | 0.4501 | 0.023 |
| Min dist—root to a split node | − 0.0446 | **0.2737** | **− 0.49169** | − 0.02017 | 0.0257 |
| *p* | < .0001 | **< 0.0001** | **< 0.0001** | 0.0662 | 0.0192 |
| Max dist—root to a split node | − 0.00168 | **0.20198** | **− 0.24517** | − 0.01897 | 0.01233 |
| *p* | 0.8781 | **< 0.0001** | **< 0.0001** | 0.084 | 0.2614 |
| Avg dist—root to a split node | − 0.03627 | **0.27154** | **− 0.46267** | − 0.02348 | 0.02474 |
| *p* | 0.001 | **< 0.0001** | **< 0.0001** | 0.0324 | 0.0242 |
| Min dist—root to the max split node | − 0.01082 | 0.17919 | **− 0.34624** | − 0.00836 | 0.02102 |
| *p* | 0.3242 | <0.0001 | **< 0.0001** | 0.4466 | 0.0555 |
| Max dist—root to the max split node | 0.00424 | 0.01456 | 0.01553 | − 0.00165 | 0.01453 |
| *p* | 0.6995 | 0.1848 | 0.1571 | 0.8808 | 0.1856 |
| Avg dist—root to the max split node | − 0.00261 | 0.08673 | − 0.14565 | − 0.00579 | 0.01751 |
| *p* | 0.8119 | <0.0001 | <0.0001 | 0.5978 | 0.1107 |
| Min dist—leaf to the last split node | 0.02556 | 0.15199 | − 0.081 | − 0.01642 | 0.01401 |
| *p* | 0.0199 | <0.0001 | <0.0001 | 0.1348 | 0.2019 |
| Max dist—leaf to the last split node | 0.00902 | − 0.02375 | 0.00217 | − 0.00539 | − 0.00693 |
| *p* | 0.4115 | 0.0305 | 0.8432 | 0.6234 | 0.5281 |
| Avg dist—leaf to the last split node | − 0.01325 | 0.19104 | − 0.23714 | − 0.01523 | 0.02352 |
| *p* | 0.2274 | <0.0001 | <0.0001 | 0.1654 | 0.0321 |

Linear one-to-one correlations between each graphical parameter and each "biological" (simulation) parameter; correlation coefficients and their *p*-values for all graphical parameters measured are shown.
Shown in bold letters are all significant correlations with $|R| > 0.2$.

the reliability of the data itself, not only due to PCR errors, but also because of the way trees are generated. As far as we know, all tree generation algorithms assume that if a mutation is shared by two different cells, then it must have occurred in a common ancestor of both cells. Thus, these algorithms do not allow for the possibility of identical mutations occurring in parallel in different "branches" of the tree. As there is no way to tell which shared mutations have indeed occurred in a common ancestor, and which shared mutations actually occurred independently, we must take the trees as they are and analyse them as such, assuming that the tree generation algorithms are at least consistent in all cases.

### METHODS

### Tree Notation and Representation

A *lineage tree* is a rooted tree where nodes correspond to B cell receptor genes. For two nodes $u$ and $v$, we say that $v$ is a daughter of $u$ if the cell corresponding to $v$ is a mutant of the cell corresponding to $u$, which differs from $u$ by only one mutation, and is one mutation further than $u$ away from the original (germline) gene. Two B cells with identical receptors will correspond to the same node. A lineage tree describes the maturation process of a B cell at a certain moment of observation—it consists only of the cells that were sampled at that moment and their ancestors back to the root. The ancestors are not necessarily sampled at the time of observation. We distinguish between three kinds of nodes (Fig. 5a):

- Root—representing the original B cell (node 0).
- Leaves—representing mutant B cells, which were alive at the time of sampling and had no daughters at the time of observation (nodes 6, 11, 12, 13 and 14).
- Internal nodes—representing B cells that were produced during the maturation process, which may have been killed by selection but have a live offspring. There are two types of internal nodes: Split nodes—those with more than one daughter (node 3 and 10); and Pass-Through nodes—those with exactly one daughter (nodes 1, 2, 4, 5, 7, 8 and 9).

Since trees may come from different external sources (published experimental data, simulations, etc.), we faced the need to define a universal format for tree representation. For this purpose we chose the adjacency list format. Each node in a tree has a unique identification (id) number, satisfying the following two conditions:

$$\text{id(Root)} = 0$$

and

$$\text{id(Daughter)} > \text{id(Parent)}$$

Hence a tree is represented by a text file, where each line contains a node id followed by its daughters' id's, delimited by space(s). Lines starting with a "#" sign are considered to be comments and thus ignored by the measurement algorithm (described below). A sample file containing the adjacency-list presentation of the tree from the previous example is shown in Fig. 5b.

(a)

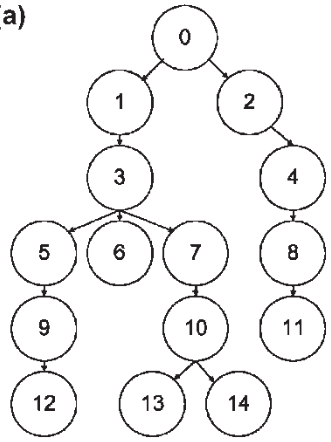

(b)

| | | | |
|---|---|---|---|
| 0 | 1 | 2 | |
| 1 | 3 | | |
| 2 | 4 | | |
| 3 | 5 | 6 | 7 |
| 4 | 8 | | |
| 5 | 9 | | |
| 7 | 10 | | |
| 8 | 11 | | |
| 9 | 12 | | |
| 10 | 13 | 14 | |

FIGURE 5 A sample tree (a), with the nodes marked by their "id" numbers; the adjacency list that corresponds to the tree is shown in (b).

### Tree Measurement

In this section we define the graphical parameters to be measured on the tree. *A priori* we measured all the graphical properties we could define on the trees, as we did not know for certain which measure would best correlate with biological parameters. We show in the "results" section which of the parameters seem to express some information of interest. Note that, for quite a few of these properties, the maximum, minimum and average values *per tree* may be measured; while it is obvious that minimum values are often trivial, we have again measured all possible properties and then looked for the ones which best correlate with biological parameters. The complete list of parameters measured is the following.

- Number of nodes—total number of nodes, number of leaves, internal nodes, pass-through nodes.
- Path length (root-to-leaf distance)—min, max, average (over all leaves in the tree).
- Outgoing degree (number of daughters per node)—min, max, average, average excluding pass-through nodes, root's degree.
- Distance from root to the first split node (trunk length)—if root's degree is 1; otherwise this distance equals zero.
- Distance between leaf and the nearest split node—min, max, average.
- Distance between leaf and the first (closest to the root) split node—min, max, average.
- Distance from root to split node (on each path to a leaf, not considering the root itself)—min, max, average.
- Distance from root to the maximal (in terms of outgoing degree) split node—min, max, average.
- Distance between two adjacent split nodes—min, max, average.

We developed a computer program that reads a tree in the format described above and measures the graphical parameters, creating a text report. As stated above, a lineage tree describes the process at the specific moment of observation. Thus, in an experimentally obtained tree, only those cells that were sampled are represented. Usually the percentage of the germinal centre cells that are sampled is not very high. In order to neutralize the effects of sampling, the above parameters have to be additionally scaled (divided) by number of nodes (total) or by the number of leaves. In the results section we further discuss this issue with respect to experimental tree measurement. For the time being it will suffice to note that for most of the properties given in the list above, three values were measured—unscaled, scaled by number of nodes (total) and scaled by number of leaves.

### Simulation of Germinal Centre Lineage Trees

The model of the affinity process implemented by our simulation ("tree generator") is very simple, yet it captures the main features of the process (Fig. 6). Our model considers a single population of B cells consisting of several clones (cells with different antigen receptors). A B cell's receptor is represented by a 512-bit string. A certain amount of antigen is available at the beginning of the simulation, where the antigenic epitope is represented by a 512-bit string as well. The affinity of a B cell is given by a (normalised) number of non-coinciding bits in the cell's receptor and the antigen (actually computed by applying the logical function XOR to the two strings bitwise). In every simulated "time step", each cell in the population can divide into two daughter cells, each one of which may undergo mutation according to the mutation parameters. The probability of the cell to divide and mutate depends on the affinity of its receptor to the antigen, population size (relatively to the maximum allowed population size) and the amount of available antigen. A newborn cell can either immediately die due to lethal mutation, divide and mutate again, or undergo selection, whichever should happen according to the simulation parameters. As a result of selection, a cell either dies, or survives. This decision is taken according to the cell's affinity and selection parameters. Each successful selection event consumes one antigen unit. The process stops whenever the antigen is exhausted or after a specified number of time steps. Several clones may develop simultaneously in the simulation. Each clone originates from a different B cell in the initial population. A lineage tree is produced for each clone that survives to the end of the simulation.

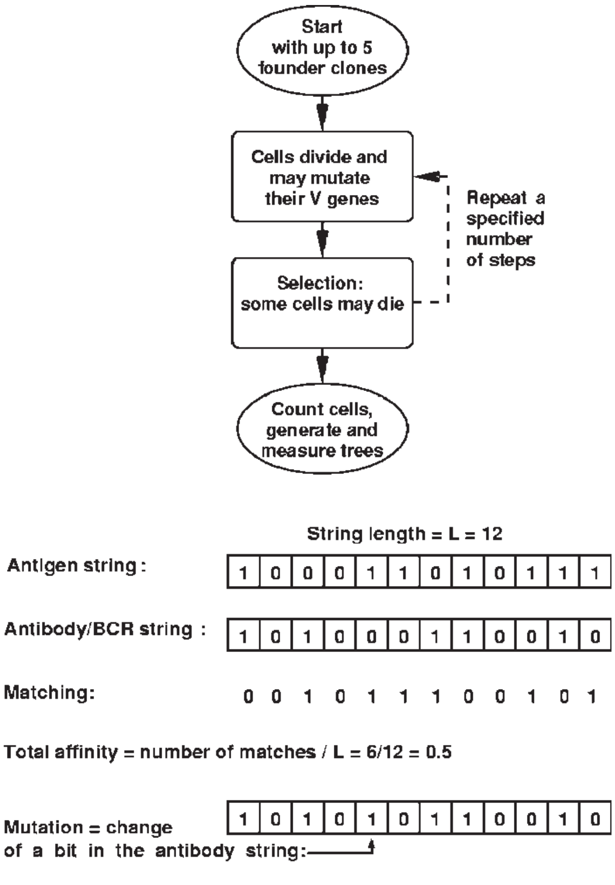

FIGURE 6 The simulation algorithm.

### Acknowledgements

The authors gratefully acknowledge useful discussions with M. Shlomchik, P. Watts, M. Weigert and S. Litwin, who kindly lent us the CLONE program, and with T. Manser and M. Golumbic; and help with statistical analyses by Ms. Rachel Levi-Drummer.

Supported in part by Israel Science Foundation grant number 759/01-1, The Yigal Alon Fellowship, and the Bar-Ilan University internal grant (to R.M.) and the Biotechnology and Biological Sciences Research Council (BBSRC) SAGE initiative (to DDW).

### References

Cowell, L.G. and Kepler, T.B. (2000) "The nucleotide-replacement spectrum under somatic hypermutation exhibits microsequence dependence that is strand-symmetric and distinct from that under germline mutation", *J. Immunol.* **164**, 1971–1976.

Diaz, M. and Casali, P. (2002) "Somatic immunoglobulin hypermutation", *Curr. Opin. Immunol.* **14**, 235–240.

Dorner, T., Foster, S.J., Brezinschek, H.P. and Lipsky, P.E. (1998) "Analysis of the targeting of the hypermutational machinery and the impact of subsequent selection on the distribution of nucleotide changes in human VHDJH rearrangements", *Immunol. Rev.* **162**, 161–171.

Dunn-Walters, D.K., Boursier, L., Ciclitira, P.J. and Spencer, J. (1997a) "Immunoglobulin genes from human duodenal and colonic plasma cells are mutated", *Biochem. Soc. Trans.* **25**, 324S.

Dunn-Walters, D.K., Isaacson, P.G. and Spencer, J. (1997b) "Sequence analysis of human IgVH genes indicates that ileal lamina propria plasma cells are derived from Peyer's patches", *Eur. J. Immunol.* **27**, 463–467.

Dunn-Walters, D.K., Dogan, A., Boursier, L. and Spencer, J. (1998) "Base-specific sequences that bias somatic hypermutation deduced by analysis of out-of-frame human IgVH genes", *J. Immunol.* **160**, 2360–2364.

Foster, S.J., Dorner, T. and Lipsky, P.E. (1999) "Somatic hypermutation of VkJk rearrangements: targeting of RGYW motifs on both DNA strands and preferential selection of mutated codons within RGYW motifs", *Eur. J. Immunol.* **29**, 4011–4021.

Honjo, T., Kinoshita, K. and Muramatso, M. (2002) "Mechanism of class switch recombination: linkage with somatic hypermutation", *Ann. Rev. Immunol.* **20**, 165–196.

Jacob, J. and Kelsoe, G. (1992) "*In situ* studies of the primary immune response to (4-hydroxy-3-nitrophenyl) acetyl. II. A common clonal origin for periarteriolar lymphoid sheath-associated foci and germinal centers", *J. Exp. Med.* **176**, 679–687.

Jacob, J., Kelsoe, G., Rajewsky, K. and Weiss, U. (1991) "Intraclonal generation of antibody mutants in germinal centres", *Nature* **354**, 389–392.

Jacob, J., Przylepa, J., Miller, C. and Kelsoe, G. (1993) "*In situ* studies of the primary immune response to (4-hydroxy-3- nitrophenyl)acetyl. III. The kinetics of V region mutation and selection in germinal centre B cells", *J. Exp. Med.* **178**, 1293–1307.

Kelsoe, G. (1996) "Life and death in germinal centers", *Immunity* **4**, 107–111.

Kepler, T.B. and Perelson, A.S. (1993) "Cyclic re-entry of germinal centre B cells and the efficiency of affinity maturation", *Immunol. Today* **14**, 412–415.

Kesmir, C. and de Boer, R.J. (1999) "A mathematical model on germinal centre kinetics and termination", *J. Immunol.* **163**, 2463–2469.

Kim, N., Bozek, G., Lo, J.C. and Storb, U. (1999) "Different mismatch repair deficiencies all have the same effects on somatic hypermutation: Intact primary mechanism accompanied by secondary modifications", *J. Exp. Med.* **190**, 21–30.

Kocks, C. and Rajewsky, K. (1988) "Stepwise intraclonal maturation of antibody affinity through somatic hypermutation", *Proc. Natl Acad. Sci.* **85**, 8206–8210.

Manser, T. (1989) "Evolution of antibody structure during the immune response", *J. Exp. Med.* **170**, 1211–1230.

Michael, N., Martin, T.E., Nicolae, D., Kim, N., Padjen, K., Zhan, P., Nguyen, H., Pinkert, C. and Storb, U. (2002) "Effects of sequence and structure on the hypermutability of immunoglobulin genes", *Immunity* **16**, 123–134.

Monson, N.L., Dorner, T. and Lipsky, P.E. (2000) "Targeting and selection of mutations in human Vλ- rearrangements", *Eur. J. Immunol.* **30**, 1597–1605.

Neuberger, M.S., Ehrenstein, M.R., Klix, N., Jolly, C.J., Yelamos, J., Rada, C. and Milstein, C. (1998) "Monitoring and interpreting the intrinsic features of somatic hypermutation", *Immunol. Rev.* **162**, 107–116.

Neuberger, M.S., Ehrenstein, M.R., Rada, C., Sale, J., Batista, F.D., Williams, G. and Milstein, C. (2000) "Memory in the B cell compartment: antibody affinity maturation", *Phil. Trans. R. Soc. Lond. B Biol. Sci.* **1395**, 357–360.

Oprea, M. and Kepler, T.B. (1999) "Genetic plasticity of V genes under somatic hypermutation: Statistical analyses using a new resampling-based methodology", *Gen. Res.* **9**, 1294–1304.

Oprea, M. and Perelson, A.S. (1997) "Somatic mutation leads to efficient affinity maturation when centrocytes recycle back to centroblasts", *J. Immunol.* **158**, 5155–5162.

Schiaffella, E., Seghal, D., Anderson, A.O. and Mage, R.G. (1999) "Gene conversion and hypermutation during diversification of VH sequences in developing splenic germinal centres of immunized rabbits", *J. Immunol.* **162**, 3984–3995.

Seghal, D., Schiaffella, E., Anderson, A.O. and Mage, R.G. (1998) "Analysis of single B cells by PCR reveals rearranged VH with germline sequences in spleen of immunized rabbits: implications for B cell repertoire maintenance and renewal", *J. Immunol.* **161**, 5347–5356.

Seghal, D., Schiaffella, E., Anderson, A.O. and Mage, R.G. (2000) "Generation of heterogeneous rabbit anti-DNP antibodies by gene conversion and hypermutation of rearranged VL and VH genes during clonal expansion of B cells in splenic germinal centres", *Eur. J. Immunol.* **30**, 3634–3644.

Seghal, D., Obiakor, H. and Mage, R.G. (2002) "Distinct clonal diversification patterns in young appendix compared to antigen-specific splenic clones", *J. Immunol.* **168**, 5424–5433.

Shannon, M. and Mehr, R. (1999) "Reconciling repertoire shift with affinity maturation: The role of deleterious mutations", *J. Immunol.* **162**, 3950–3956.

Shlomchik, M., Watts, P., Weigert, M. and Litwin, S. (1998) "Clone: a Monte-Carlo computer simulation of {B} cell clonal expansion, somatic mutation and antigen-driven selection", *Curr. Top. Microbiol. Immunol.* **229**, 173–197.

Song, H., Nie, X., Basu, S. and Cerny, J. (1998) "Antibody feedback and somatic mutation in B cells: regulation of mutation by immune complexes with IgG antibody", *Immunol. Rev.* **162**, 211–218.

Song, H., Basu, S., Nie, X. and Cerny, J. (1999) "Regulation of VH gene repertoire and somatic mutation in germinal centre B cells by passively administered antibody", *Immunology* **98**, 258–266.

Spencer, J., Dunn, M. and Dunn-Walters, D.K. (1999) "Characteristics of sequences around individual nucleotide substitutions in IgVH genes suggest different GC and AT mutators", *J. Immunol.* **162**, 6596–6601.

Sulzer, B., van Hemmen, L., Neumann, A.U. and Behn, U. (1993) "Memory in idiotypic networks due to competition between proliferation and differentiation", *Bull. Math. Biol.* **55**, 1133–1182.

Vora, K.A., Tumas-Brundage, K. and Manser, T. (1999) "Contrasting the *in situ* behaviour of a memory B cell clone during primary and secondary immune responses", *J. Immunol.* **163**, 4315–4327.

Wabl, M., Cascalho, M. and Steinberg, C. (1999) "Hypermutation in antibody affinity maturation", *Curr. Opin. Immunol.* **11**, 186–189.

Winter, D.B. and Gearhart, P.J. (1998) "Dual enigma of somatic mutation of immunoglobulin variable genes: targeting and mechanism", *Immunol. Rev.* **162**, 89–96.

### APPENDIX: THE FORMAT OF TREES FOR ANALYSIS

The tree analysis program is under development, and will be available upon request when completed. In the meantime we are willing to analyse data. Updates will be posted on our web site: http://repertoire.ls.biu.ac.il/TREES/, which also contains some demonstrations of the method and instructions on the format in which the data should be submitted.

